# Adipose-derived stem cells in immune-related skin disease: a review of current research and underlying mechanisms

**DOI:** 10.1186/s13287-023-03561-8

**Published:** 2024-02-08

**Authors:** Tianyi Sun, Cheng Zhou, Feng Lu, Ziqing Dong, Jianhua Gao, Bin Li

**Affiliations:** The Department of Plastic and Cosmetic Surgery, Nanfang Hospital, Southern Medical University, 1838 Guangzhou North Road, Guangzhou, 510515 Guangdong China

**Keywords:** Adipose-derived stem cells, Immune-related skin disease, Immune cells, Paracrine mechanisms, Autoimmune

## Abstract

Adipose-derived stem cells (ASCs) are a critical adult stem cell subpopulation and are widely utilized in the fields of regenerative medicine and stem cell research due to their abundance, ease of harvest, and low immunogenicity. ASCs, which are homologous with skin by nature, can treat immune-related skin diseases by promoting skin regeneration and conferring immunosuppressive effects, with the latter being the most important therapeutic mechanism. ASCs regulate the immune response by direct cell–cell communication with immune cells, such as T cells, macrophages, and B cells. In addition to cell–cell interactions, ASCs modulate the immune response indirectly by secreting cytokines, interleukins, growth factors, and extracellular vesicles. The immunomodulatory effects of ASCs have been exploited to treat many immune-related skin diseases with good therapeutic outcomes. This article reviews the mechanisms underlying the immunomodulatory effects of ASCs, as well as progress in research on immune-related skin diseases.

**Supplementary Information:**

The online version contains supplementary material available at 10.1186/s13287-023-03561-8.

## Introduction

Immune-related skin diseases are a type of damage caused by disorders of the immune system, which are characterized by overactivated immune cells, high levels of pro-inflammatory factors, and a series of complex immune responses. Clinical symptoms of immune-related skin disease include fibrosis, redness, swelling, itching, and dandruff of the skin [1]. Topical and oral immunosuppressive drugs are the most widely used to treat immune-associated skin diseases, and new, more targeted approaches to treatment are being developed, but existing methods are still limited by the long course of treatment, limited efficacy, and significant expense [2]. ASCs are a type of mesenchymal stem cells (MSCs) derived from adipose tissue, also originate from the mesoderm, and are closely related to the skin. ASCs can thus be used to replenish and treat damaged skin tissue. Compared to other MSCs, ASCs have a potent immunosuppressive effect, which is a significant treatment advantage for immune-related skin diseases [3]. ASCs undergo cell–cell interactions with a variety of immune cells, including T cells, macrophages, B cells, dendritic cells (DCs), and natural killer cells (NK cells). Paracrine mechanisms of ASCs also indirectly regulate the immune system via secretion of cytokines, growth factors, anti-inflammatory mediators, active enzymes, and extracellular vesicles (EVs). ASC-secreted EVs and apoptotic ASCs have similar therapeutic benefits to viable ASCs, suggesting that the paracrine mechanisms of ASCs are central to their immunomodulatory effects [4]. The present review describes ASC immunomodulatory mechanisms that contribute to their therapeutic effects in treatment of immune-related skin diseases and summarizes the progress of preclinical research and clinical application of ASC treatment of autoimmune skin diseases. In addition, the challenges and adverse reactions faced by ASCs in clinical application are also mentioned.

## ASC overview

ASCs are a type of MSC derived from adipose tissue. Compared with bone marrow mesenchymal stem cells (BM-MSCs), umbilical cord MSCs, and other MSCs, ASCs display unique advantages with respect to immunomodulation [5]. They can be harvested repeatedly, and in large quantities, from subcutaneous tissue by liposuction under anesthesia. Liposuction is also more comfortable than the painful bone marrow aspiration process and is more desirable because more stem cells can be harvested from the same amount of tissue; the process even has esthetic effects [6]. Additionally, studies gradually show that ASCs facilitate generation of keratinocytes and the secretome, resulting in improved skin regeneration [7]. Generally, ASCs have outstanding immunomodulatory and skin repair-promoting effects, which makes them an ideal candidate for stem cell therapy of immune-related skin diseases.

ASCs have many abilities favorable for the treatment of immune-related skin diseases, such as direct differentiation into skin cells and release of secretomes that promotes skin growth, together with their immunoregulatory effects. However, the latter plays a central role in the therapeutic effects. This is because the restoration of the disordered immune system is the cornerstone for treatment of immune-related skin diseases [8].

The immunomodulatory effects of ASCs are due to both cell–cell interactions and paracrine mechanisms, which affect different targets. Cell–cell interactions with ASCs are most common in T cells, macrophages, and B cells, which are also related to the pathophysiological characteristics of pathogenic immune cells in immune-related skin diseases. Paracrine effects of ASCs affect the fate and function of immune cells and the immune microenvironment via multiple bioactive factors in secretomes, and these paracrine effects are essential to the immunomodulatory role of ASCs [9].

## Immunomodulatory effect of ASCs in immune-related skin diseases

**Fig. 1:**
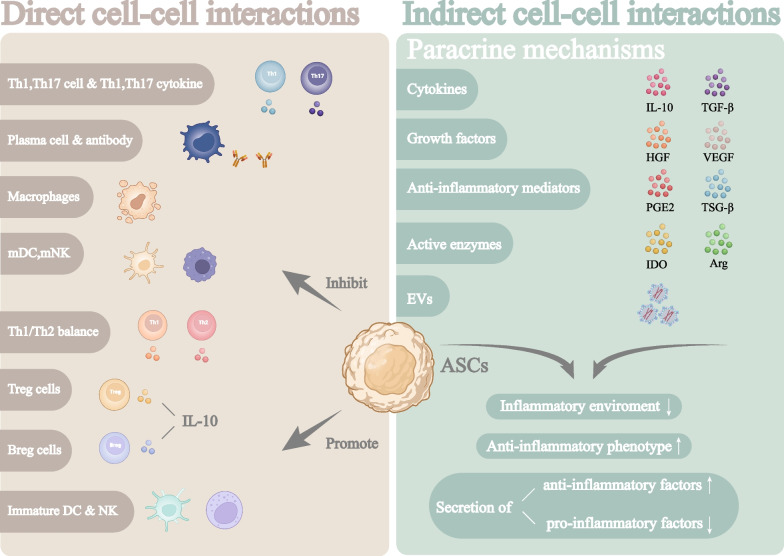
Direct and indirect (paracrine mechanisms) interactions of ASCs during immunomodulation. This schematic highlights the direct cell–cell interactions of ASCs, such as their effects on T lymphocytes, macrophages, B lymphocytes, DCs and NK cells, and the factors secreted by these cells. Additionally, the paracrine effects of cytokines, growth factors, anti-inflammatory mediators, active enzymes, and EVs play an important role in regulating the immune environment and immune cell function. Created with BioRender.com

Compared to other MSCs, ASCs have the strongest immunomodulatory properties, suggesting the therapeutic utility of ASCs in immune-related skin diseases [3]. Because ASCs and skin are both derived from the mesoderm and are anatomically closely related, ASCs alleviate immune-related skin diseases by both promoting regeneration of damaged skin and suppressing autoimmunity. However, the immunomodulatory effects of ASCs are the most studied (Fig. 1).

According to the type of target, the immunomodulatory effects of ASCs can be divided into direct cell–cell interactions and indirect paracrine mechanisms (Table 1) [10].

**Table 1:** Mechanisms of ASC immunomodulatory effects

Target	Components	Mechanisms	References
T cells	*T*_reg_ cells	Promote *T*_reg_ cells differentiation and proliferation	[11–14]
	*T*_H_1/*T*_H_2	Maintain *T*_H_1/T_H_2 balance by regulating their secretory phenotype	[15, 16]
	T_H_17	Inhibit secretion of T_H_17 cytokines and induce IL-10 production	[17–20]
	CTL	Inhibit proliferation and CD8 receptor expression of CTL	[21, 22]
Macrophages	Pro-inflammatory phenotype	Inhibit differentiation and infiltration of pro-inflammatory phenotype	[23, 24]
	Anti-inflammatory phenotype	Induce differentiation to anti-inflammatory phenotype	[25–28]
B cells	Activated B cells	Inhibit differentiation and antibody production of active B cells	[29, 30]
	B_reg_ cells	Support B_reg_ cells proliferation and differentiation	[31, 32]
NK cells	–	Inhibit NK cells proliferation and their secretory phenotype	[33, 34]
DCs	–	Inhibit DCs maturation and costimulatory signals	[35, 36]
Cytokines	IL-10	Inhibit activation of multiple immune cell types	[37–40]
	TGF-β	Induce T_reg_ cells and anti-inflammatory macrophages Inhibit DCs and MLRs	[40, 41]
Growth factors	HGF	Inhibit T_H_1 cells and T_H_17 cells Suppress fibrosis	[42–45]
	VEGF	Promote angiogenesis and lymphangiogenesis Inhibit effector immune cells and promote anti-inflammatory cells	[46, 47]
Anti-inflammatory mediators	PGE_2_	Induce IL-10 production and T_reg_ cells Inhibit NK cells	[48–53]
	TSG-6	Promote anti-inflammatory macrophages and inhibit pro-inflammatory immune cells	[54–58]
Active enzymes	IDO	Mainly inhibit T cells function	[3, 59–62]
	NOS	Promote pro-inflammatory macrophages	[63, 64]
	Arg-1	Promote anti-inflammatory macrophages	[63–65]
CM	–	Inhibit immune cells infiltration and their secretory phenotype	[66–70]
EVs	–	Inhibit inflammation	[71, 72]
	Exos	Induce T_reg_ cells and anti-inflammatory macrophages Decrease inflammatory cytokines Transfer mitochondrial components	[73–75]

Treg cells, Regulatory T cells; Th1, Type 1 helper T cells; Th2, Type 2 helper T cells; Th17, Type 17 helper T cells; CTL, Cytotoxic T lymphocyte; Breg cells, Regulatory B cells; IL, Interleukin; TGF, Transforming growth factor; MLRs, Mixed lymphocyte reactions; HGF, Hepatocyte growth factor; VEGF, Vascular endothelial growth factor; PGE_2_, Prostaglandin E_2_; TSG-6, Tumor necrosis factor alpha induced protein-6; IDO, Indoleamine-pyrrole 2,3,-dioxygenase; NOS, Nitric oxide synthase; Arg, Arginase; CM, Conditioned medium; Exos, Exosomes

### Direct cell–cell interactions

Direct cell–cell interactions between ASCs and immune cells are general. Studies confirm that ASCs regulate immune cells, including T cells, macrophages, B cells, and other immune cells, to promote immune tolerance. These direct cell–cell interactions occur in the context of immune-related skin diseases. ASCs inhibit the overactivated autoimmune responses that cause immune-related skin diseases. Their immunomodulatory effects include regulating immune cell phenotypic and secretory function, and promoting a shift from a pro-inflammatory state to a static or anti-inflammatory state.

### T lymphocytes

T cell immune dysregulation involves predominantly T_H_1/T_H_17 polarization and the inability of T_reg_ cells to repress the immune response, which is implicated in immune-related skin diseases [76]. Prior studies have demonstrated that ASCs downregulate pro-inflammatory factors by affecting the expression of T cell subset transcription factors and promoting differentiation of T_H_0 cells into T_H_1, T_H_2, T_H_17, and FoxP3^+^ T_reg_ cells to regulate adaptive immunity. Additionally, ASCs decrease the number of activated T cells by arresting the cell cycle in the G0–G1 phase [49]. Compared to ASCs derived from healthy donors, ASCs derived from patients with immune-related diseases can also affect the function and phenotype of T cells, which makes the latter a reliable source for autologous applications [77].

The immunosuppressive effects of ASCs are regulated by maintaining the T_H_1/T_H_2 balance and regulating T cell secretory phenotype. ASCs inhibit effector T_H_1 cells in autoimmune diseases and restore the T_H_1/T_H_2 balance by promoting T_H_1 cytokines and inhibiting T_H_2 cytokines [15, 16]. ASCs and ASC-derived cells attenuate atopic dermatitis (AD) by suppressing inflammation associated with the T_H_1/T_H_2 response [78, 79]. Additionally, prior studies have demonstrated that ASCs suppress development of lupus dermatitis by suppressing T_H_1/T_H_2 ratio and maintaining their secretome balance [80].

ASCs also inhibit T_H_17 cell differentiation and secretion of IL-17 factors to prevent the pro-inflammatory effects of T_H_17 cells [20]. However, prior studies have also identified that ASCs promote differentiation of activated T cells into T_H_17 cells in some inflammatory environments, suggesting that development of future ASC-based immunotherapies should carefully consider these complex and detailed molecular interactions [18]. Psoriasis is a typical T_H_17-driven immune-related skin disease, and its clinical manifestations are alleviated by ASCs [81, 82]. Subcutaneous injection of ASCs also ameliorates AD by downregulating IL-17 secretion by T_H_17 cells [83].

ASCs also respond to the stimuli of pro-inflammatory factors and secrete specific factors to induce formation of T_reg_ cells, which are recruited to the skin and resolve inflammation associated with multiple autoimmune skin diseases and promote tissue healing in these contexts [12, 84]. Surprisingly, ASC-EVs also induce formation of T_reg_ cells. A prior study found that ASC-EVs induce peripheral blood mononuclear cell (PBMC) apoptosis and suppress PBMC and CD4^+^ T cell proliferation [14].

Transplantation of ASCs ameliorated autoimmune pathogenesis in a mouse systemic lupus erythematosus (SLE) model by modulating the balance between T_reg_ cells and T_H_17 cells [85]. ASC-induced T_reg_ cell amplification significantly alleviates the clinical and pathological changes of immune-related skin diseases and promotes immune tolerance to the skin barrier.

### Macrophages

The immunomodulatory effect of ASCs on macrophages is promoted by promoting the transition from the pro-inflammatory phenotype (“classically activated” or “M1” macrophages) to the anti-inflammatory phenotype (“alternatively activated” or “M2” macrophages). As classic immune cells, pro-inflammatory macrophages promote differentiation of T_H_1 cells and release pro-inflammatory factors such as tumor necrosis factor-α (TNF-α), monocyte chemoattractant protein-1, IL-6, and NOS [63]. ASCs relieve tissue inflammation by inhibiting infiltration of pro-inflammatory macrophages and secreting PGE_2_ to promote polarization to the anti-inflammatory phenotype [23]. The inhibition of ASCs on pro-inflammatory macrophages synergizes with the anti-proliferative properties of ASCs to suppress abnormal cell hyperplasia induced by chronic inflammation [86].

Anti-inflammatory macrophages secrete anti-inflammatory factors such as IL-10 and Arg-1. The two arginine metabolic pathways catalyzed by Arg-1 and NOS arrest each other, which has essential functions in regulating macrophage polarization [63]. ASCs secrete IL-10 to activate the STAT3/Arg-1 pathway, thus inducing differentiation into an anti-inflammatory phenotype [28]. A prior study of remote ASC transplantation demonstrated that tissue infiltration of anti-inflammatory macrophages is increased by intervention, suggesting that the secretory function of ASCs likely has essential regulatory roles in macrophages [26]. Apoptotic ASCs also promote Arg-1 activity and decrease nitric oxide levels in macrophages [25].

Pro-inflammatory macrophages promote the development of psoriasis by maintaining T_H_1 cytokine levels, which could be inhibited by ASCs [87]. In the bleomycin-induced systemic scleroderma (SSc) mouse model, ASCs alleviate skin fibrosis by suppressing infiltration of macrophages into the dermis [88, 89]. Moreover, AD induced by skin infection with *Staphylococcus aureus* in mice is alleviated via enhancing the phagocytic activity of macrophages by ASCs [90].

### B lymphocytes

Multiple studies have identified that ASCs affect B cell proliferation and differentiation, with complex effects depending on the inflammatory environment: At high levels of inflammation, ASCs inhibit B cell proliferation, while at low levels of inflammation, ASCs support formation of B_reg_ cells [91]. ASCs suppress over-proliferation of B cells in postinfectious inflammatory state, which is mediated by galectin-9 and B cell activating factor, to modulate the B cell immune response in vivo [30]. ASCs also inhibit production of pathogenic plasma cells and autoreactive antibodies in multiple immune-related skin diseases, such as SLE, SSc, and psoriasis. Furthermore, ASCs increase B_reg_ cell formation, prompting secretion of TGF-β1 and IL-10 to inhibit inflammation in an in vitro model co-cultured with B cells [31]. Autoreactive plasma cells elicit production of pathogenic antibodies and deposition of immune complexes in SLE [92]. Intravenous injection of ASCs alleviates autoimmunity in this context by inducing B_reg_ cell expansion and decreasing effector B cells in the SLE mouse model [93].

### Other immune cells

ASCs also interact with immune cells such as NK cells and DCs to exert immunosuppressive effects. ASCs significantly decrease the number of CD49b^+^ NK cells, decrease production of interferon-γ (IFN-γ), and increase production of IL-4 and IL-10 [34]. An additional study demonstrated that ASCs secrete high levels of IDO, IL-10, and TGF-β1 to affect NK cell phenotype, further supporting the immunosuppressive effects of ASCs on NK cells [33].

Additionally, ASCs affect DC maturation by altering the phenotypic profile of classical markers and decreasing production of pro-inflammatory cytokines while increasing the concentration of immunosuppressive factors [36]. ASC-derived Exosomes (Exos) also have regulatory effects on DCs, not only decreasing DC surface marker expression but also inhibiting DC release of inflammatory factors, indicating that the ASC-derived secretome is potentially an important modulator of immune-related skin diseases regulated in part by DCs [35]. ASCs inhibit DC maturation and secretion of cytokines via multiple regulatory mechanisms. DCs are the primary source of pathogenic TNF-α and IL-23 in psoriasis, suggesting ASC implantation as a potential therapeutic modality for this condition [94].

### Indirect cell–cell interactions (paracrine mechanisms)

ASCs secrete diverse bioactive factors such as cytokines, growth factors, anti-inflammatory mediators, and EVs to modulate the immune response in many autoimmune diseases (Table 1) [10]. These ASC-derived immunomodulatory factors can be secreted in vivo through direct transplantation of ASCs, or enriched in ASCs derivatives obtained by culture in laboratory for further use [95]. Some preclinical trials of ASCs in the treatment of immune-related skin diseases are summarized here. ASCs have been found to alleviate tissue inflammation in animal models mainly by regulating many secretome [22, 78–83, 85, 89, 90, 93, 96–117]. The preclinical studies of the immunomodulatory effect of ASCs in immune-related skin disease are summarized in Additional file 1: Table S1.

### Cytokines

IL-10 and TGF-β are prominent anti-inflammatory factors contributing to the therapeutic effects of ASCs. IL-10 has crucial immunomodulatory roles in immune-related skin diseases, suppressing excessive inflammatory responses and promoting tissue regeneration [37]. IL-10 inhibits release of pro-inflammatory factors and suppresses pro-inflammatory effects of many immune cell types [38]. Prior studies have identified that ASCs secrete IL-10 to induce polarization of immune cells into anti-inflammatory phenotypes [28]. Further, systemic ASC infusion increases spleen-derived IL-10 expression and release, exerting systemic anti-inflammatory effects [39]. Further, ASC-EVs have anti-inflammatory and pro-angiogenic effects that alleviate tissue damage, which is potentially due to IL-10 harbored in EVs [40].

TGF-β regulates immune cell function, for example by inhibiting the expansion of CD8^+^ cytotoxic T cells, and also promoting development of both CD4^+^ T_H_17 cells and T_reg_ cells, with the latter role potentially being more functionally significant [41]. Additionally, the TGF-β pathway plays a major role in B_reg_ cell induction and the immunomodulatory properties of B_reg_ cells [32]. DCs are also regulated by ASC-derived TGF-β1, inhibiting DC maturation and expression of surface markers [118].

ASCs increase IL-10 and TGF-β levels to inhibit pathological inflammation in a variety of immune-related skin diseases. For example, ASC extract alleviates pathological AD symptoms due to its content of IL-10 and TGF-β1 [107]. ASC-derived IL-10 increase the proportion of CD4 + FoxP3 + cells and ameliorates immunologic dysfunction in the SLE mouse model [103]. However, TGF-β, a fibrogenic factor, contributes to skin sclerosis of SSC and chronic sclerodermatous graft-versus-host disease (Scl-GvHD) as well. ASCs also inhibit excessive TGF-β levels and subsequent activation of downstream signaling pathways in fibrotic dermatoses to improve skin texture [98, 117].

### Growth factors

HGF is a pleiotropic growth factor with antifibrotic and immunomodulatory effects that are due not only to its inhibitory effects on T_H_1 and T_H_17 cells but also promotion of T_reg_ cells [42, 45]. ASCs secrete high levels of HGF to alleviate inflammation and promote tissue regeneration. A prior study identified that ASC-rich stromal vascular fraction (SVF) expressed high HGF levels to inhibit inflammation and fibrosis [44]. Further, ASC-derived HGF promotes tissue vascularization and repair [43]. These findings suggest that ASC-secreted HGF could be advantageous in treatment of tissue damage caused by inflammation. ASC-derived HGF exerts the anti-inflammatory and antifibrotic effects in improving skin sclerosis in SSc [119].

VEGF is widely studied and has immunomodulatory effects in addition to its well-known roles in promoting angiogenesis and increasing vascular permeability. VEGF secreted by ASCs can both downregulate expression of adhesion molecules in endothelial cells to hinder immune cell adhesion and support the expansion of T_reg_ cells and anti-inflammatory macrophage infiltration by promoting angiogenesis [46, 120]. VEGF also directly modulates immune cells, for example by inhibiting effector T cell function, hindering differentiation and activation of DCs, and increasing recruitment of T_reg_ cells [121]. In immune-related skin diseases, increased VEGF levels are primarily related to promotion of vascularization and repair of damaged tissue. Many preclinical studies have demonstrated that local angiogenesis of lesions was significantly improved by ASC-derived VEGF and that ulcers and skin fibrosis caused by SSc were also alleviated [96, 101].

### Anti-inflammatory mediators

Other anti-inflammatory mediators affected by ASCs include PGE_2_ and TSG-6. The immunosuppressive properties of PGE_2_ are primarily manifested by inducing macrophages to secrete IL-10, inhibiting DC maturation and decreasing NK cell cytotoxicity [122]. The effect of ASC-secreted PGE_2_ inhibits T cell proliferation and induces formation of T_reg_ cells [49, 52]. Moreover, ASC-derived PGE_2_ modulates differentiation of myeloid cells toward anti-inflammatory profiles [50]. Co-culture of ASCs with T cells derived from SSc and SLE patients revealed that ASCs suppressed expansion of CD4^+^ and CD8^+^ T cells by secreting PGE_2_ and kynurenines [22]. ASCs also significantly activates the COX-2/PGE2 cascade to inhibit growth of abnormal fibroblasts in keloids, which exerts an anti-fibrotic effect as well [52].

TSG-6, which is produced by TNF-stimulated ASCs, attenuates the immune response and promotes tissue regeneration in multiple immune-related skin diseases [54]. ASCs secrete TSG-6 to inhibit pro-inflammatory cytokines, including IL-1β, IL-6, and TNF-α [55]. Intraperitoneally injection of ASC enhances TSG-6 levels to induce macrophage polarization from pro-inflammatory phenotype to anti-inflammatory phenotype [56]. Further, TSG-6 plays an important role in ASC-EV induction of T_reg_ cells [57]. TSG-6 secreted by ASCs has essential anti-inflammatory effects in many autoimmune diseases and inflammatory injuries, which are modulated by regulating macrophage phenotypes and alleviating endoplasmic reticulum stress [56, 123]. Future research will better define the effects of TSG-6 in immune-related skin diseases.

### Active enzymes

ASCs are the most potent MSCs in immunomodulation, and their immunosuppressive effects are mediated in part by the IDO-kynurenine pathway, which regulates T cell suppression [49]. Consistently, IDO-silenced ASCs were unable to increase T_H_2 cells, and HGF expression was decreased, suggesting that their immunosuppressive effects were attenuated [61]. More in-depth studies of IDO have revealed that the two IDO subtypes, IDO1 and IDO2, have different functions. IDO1 mediates T cell suppression, while IDO2 affects B cells, functioning as a pro-inflammatory mediator of B cell responses [60].ASCs-derived IDO1 also induce macrophage polarization to the anti-inflammatory phenotype, which consequently alleviates inflammation and fibrosis [62].

Arginine metabolism is important for macrophage polarization, and its distinct metabolic pathways are catalyzed by NOS and Arg, which block each other [63]. The pro-inflammatory macrophages primarily secrete NOS, while anti-inflammatory macrophages secrete Arg. ASCs decrease the initial expression of inducible NOS and promote Arg-1 expression in infiltrating macrophages, consistent with a shift toward an anti-inflammatory phenotype [64]. Moreover, ASC-Exos transferred into macrophages induce the anti-inflammatory phenotype via transactivation of Arg-1 via STAT3 contained in vesicles [65].

T cells are central mediators of host rejection in the GvHD model, and inhibition of T cells is therefore the primary target for prolonging the survival of skin allografts. In a humanized skin allograft rejection model, ASCs suppress T cell-mediated alloreactivity by increasing IDO mRNA expression and IDO protein activity [114]. In a full-thickness skin grafts rats model treated with ASCs, NOS levels were markedly decreased, while Arg-1 and IL-10 levels were substantially increased, suggesting that the anti-inflammatory effect of ASCs could indirectly contribute to skin graft survival by regulating macrophage polarization [115].

### ASC-EVs

EVs are subcellular structures consisting of a lipid bilayer membrane encasing cytoplasm, formed by invagination of the plasma membrane, which is roughly divided into two types according to their diameter and origin, including Exos with a diameter of 50–150 nm derived from endosomes and microvesicles with a diameter of 50–500 nm derived from the plasma membrane [124]. Multiple studies have demonstrated that ASC-EVs have the same therapeutic effect as ASCs in inducing immune tolerance [95].

A prior study suggested that the anti-inflammatory effect of ASC-EVs could be related to the suppression of NF-κB-dependent inflammatory/catabolic environments [125]. Intravenous administration of ASCs enable the transfer of ASC-EVs to anti-inflammatory macrophages subsequently enhanced T_reg_ cell induction, which underlies ASC immunotherapy [72].

As subpopulations of ASC-EVs, ASC-Exos also contain Arg-1, which promotes anti-inflammatory macrophages polarization while inhibiting T cell proliferation [73]. Interestingly, ASC-Exos can effectively donate mitochondria components to macrophages, which improves macrophage mitochondrial integrity and oxidative phosphorylation, allowing resumption of macrophage metabolic and immune homeostasis and mitigating inflammatory pathology [75].

ASC-Exos were injected subcutaneously into the lesion sites of AD, which decreased expression of pro-inflammatory factors in the skin, increased ceramide synthesis, and alleviated clinical symptoms of AD [110]. Further studies have demonstrated that ASC-Exo improvement of inflammation and skin barrier function is regulated by suppressing the JAK/STAT pathway in skin lesions of AD [111]. In addition, compared to ASCs, ASC-EVs cocultured with SSc-like myofibroblasts significantly downregulate myofibroblast markers and inhibit TGF-β stimulation, further underscoring the therapeutic potential of ASC-EVs in SSc [99].

Compared with naive ASCs, ASC-EVs not only have the advantages of stable transportation and convenient storage but also contain simple and well-defined components, allowing a more precise curative effect. Further, ASC-EVs can be used as effective carriers for multiple drugs, allowing more precise drug delivery and absorption in skin lesions [126].

## Progress in clinical application of ASCs for treatment of immune-related skin diseases

**Fig. 2:**
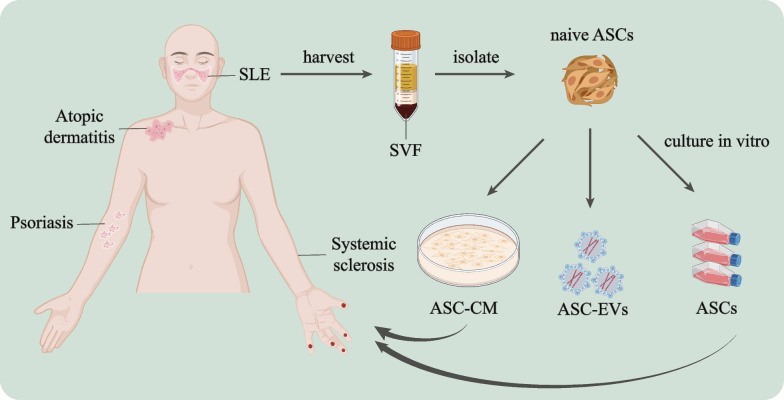
Application progress of ASCs on immune-related skin diseases. The image shows the process of harvest, isolation, and in vitro culture of ASCs from a donor, and the application of amplified ASCs and collected ASCs derivatives in the treatment of patients with immune-associated skin diseases. Created with BioRender.com

Immune-related skin diseases include a range of complex disorders that may affect all systems or have skin-only manifestations. According to immune status, they can be roughly divided into two categories: suppressed or hyper-reactive. Among these, immunodepression leads to dermatologic diseases such as Herpes zoster, Kaposi sarcoma, and fungal infection in elderly patients or patients with organ transplants, especially those who have HIV [127]. Skin disorders caused by hyper-reactive immune responses are the most common immune-related skin diseases, which include autoimmune, allergic, infectious, and tumorous skin disorders [128]. ASCs have excellent immunomodulatory effects and skin reparative effects, prompting much research into their application to immune-related skin diseases. The utility of ASCs and their cell-free derivatives as immunotherapies in immune hyper-reactive dermatosis is supported by multiple studies (Table 2) [95]. Investigators have used ASCs and ASC derivatives for treatment of SSc, psoriasis, SLE, AD, and others (Fig. 2). Both autologous and allogeneic ASCs have been used in multiple clinical trials. While autologous ASCs are considered to be safer due to their lower immunogenicity, allogeneic ASCs are more commonly used due to their increased availability and reproducibility.

**Table 2:** Clinical efficacy ASCs in immune-related skin diseases

Diseases	Study design	Efficacy	Transplant material	References
SSc	Clinical trial (*n* = 6)	Significantly improved tightening of the skin without complications	ASCs	[129]
	Phase I trial (*n* = 12)	Significantly improved hand disability, pain, blood supply, and finger edema	SVF	[139]
	Pilot study (*n* = 15)	Promotion of ulcer healing and increased capillaries in fingers	ASCs	[140]
	Pilot study (*n* = 10)	Both ASCs and fat transplantations had significant effects, but the relative utility of each strategy could not be determined	ASCs / fat transplantation	[141]
	Pilot study (*n* = 12)	Improved sclerosis, vascularization, and hand motion and strength	SVF	[142]
	Case report (*n* = 1)	Improved blood flow and prevention of further amputation	SVF	[143]
	Case report (*n* = 1)	Improved skin elasticity and vascularization	SVF (& PRP)	[144]
	Clinical trial (*n* = 62)	Improved clinical symptoms in orofacial fibrosis	ASC-enriched lipotransfer	[145]
	Clinical trial (*n* = 20)	Improvements in skin fibrosis, hand disability, Raynaud’s phenomenon, and active ulcers	SVF	[146]
	Pilot study (*n* = 18)	Improved fat graft retention in the ASC‐assisted group to correct facial atrophy	ASC-assisted fat grafting	[147]
	Phase II trial (*n* = 40)	Improved hand function in both groups over time, with no increased therapeutic efficacy of SVF relative to placebo	SVF( ±)	[134]
	Randomized controlled trial (*n* = 88)	Trends of clinical improvements in patients with diffuse cutaneous SSc	ASCs	[130]
Psoriasis	Case report (*n* = 2)	Demonstrated safety and tolerability and decreased dependence on immunosuppressant drugs	ASCs	[148]
	Case report (*n* = 1)	Safely improved symptoms with a noticeable difference in skin quality and appearance	SVF	[131]
	Case study (*n* = 1)	Completely regressed psoriasis and cleared inflammatory erythematous plaques	ASC-CM	[149]
	Pilot study (*n* = 7)	Modality is safe to use and a potential therapeutic option	ASCs	[150]
SLE	Phase I trial (*n* = 9)	Established safety of the modality and potential efficacy in decreasing disease severity	ASCs	[151]
AD	Case report (*n* = 2)	Marked improved erythematous facial lesions	ASC-Exos	[132]

*PRP* Platelet-rich plasma

As early as 2013, Scuderi et al. conducted a clinical trial using autologous ASC transplantation for SSc patients, and all six patients enrolled in the study benefited from arrest of local disease progression. In addition, they also demonstrated that neither function nor phenotype differed between ASCs derived from patient donors and healthy donors, establishing the basis for subsequent use of autologous ASCs in patients with immune-associated skin diseases [129].

Khannaden et al. reported the largest randomized clinical trial thus far. Eighty-eight patients with SSc-induced hand disability were randomly assigned to the autologous ASC group or placebo group, and changes in hand function were assessed. Compared to the control group, the ASC group had improved hand function, but the difference was not statistically significant (mean ± SD improvement in Cochin Hand Function Scale score at 48 weeks 11.0 ± 12.5 vs 8.9 ± 10.5; *P* = 0.299). Among patients enrolled in the study, hand function was most improved in patients with diffuse cutaneous SSc (dcSSc) (52% dual-response rate compared to 16% in the placebo group; nominal *P* = 0.016). The authors suggested that further clinical trials of this intervention in the context of dcSSc are warranted [130].

Other studies have explored the therapeutic potential of ASC derivatives. Comella et al. reported the first case study of intravenous SVF implantation in psoriasis. The patient benefited from a significant decrease in symptoms and skin quality appearance without safety concerns or severe adverse events [131]. Furthermore, Park et al. underscored the therapeutic potential of ASC-Exos for AD patients. Two patients with AD and refractory dupilumab facial redness were successfully improved with ASC-Exos. Importantly, this trial did not use autologous ASC-Exos, confirming that clinical application of allogeneic ASCs-Exos does not cause immune rejection, making ASCs derivatives an ideal material for allogeneic applications [132].

However, some reports suggest a dualistic function of ASCs in SSc as ASCs could function as an additional pathogenic source of pro-fibrotic myofibroblasts via adipocyte-to-myofibroblast transition, resulting in lack of therapeutic effect or even potential aggravation of SSc [133]. A prior study might support this theory. A double-blind, multicenter, phase II trial assessed the efficacy of SVF vs placebo injection into the fingers in improvement of hand disability in 40 patients, showing no additional therapeutic effects for SVF over time. The author indicated that studies of more patients with the same phenotype should be conducted to more accurately assess the benefits of ASC treatment [134].

The means for ASC induction to exert the desired immunomodulatory and therapeutic effects in different stages of different immune-related skin diseases remains a major barrier to treatment. Because the immunomodulatory effects of ASCs are regulated primarily by paracrine mechanisms, multiple strategies to regulate ASC paracrine effects have been evaluated [135]. A prior study used fibrous-engineered scaffolds to induce ASC expression of higher levels of anti-inflammatory factors via a mechanotransduction pathway [136]. Additionally, using chitosan film as a 3D culture strategy significantly affects ASC production of immunosuppressive factors in vitro through increased secretion of TGF-β and IL-10 and increased Arg activity [137]. ASC-Exos combined with hydrogels are more easily absorbed by the body, alleviating early inflammation and promoting tissue repair [138]. Thus, the combination with materials increases duration of action, induces sustained release, and changes the route of drug administration, increasing the range and efficacy of ASC applications. Although the above strategies are presently still in the research stage, their potential for clinical application is worth expecting.

## Challenges and adverse reactions during clinical application of ASCs

In recent years, the techniques used to harvest, isolate, and inject ASCs have been studied and standardized. However, challenges remain with respect to clinical application. Differences in donor age, sex, body mass index, and donor site lead to heterogeneity of ASCs, which makes treatment efficacy unpredictable. In addition, the research and clinical application of human stem cells must follow strict guidelines and pass ethical reviews, which limits clinical application of ASCs.

We cannot ignore the existence of adverse reactions associated with ASC-based therapies. Current concerns related to the clinical application of ASCs focus mainly on embolism caused by intravascular injection, and possible tumorigenicity, which gives rise to ethical problems. Although an increasing number of clinical studies have refuted these concerns, further work is needed [152–154].

Although some of the interactions between ASCs and immune cells mentioned in this review have not been confirmed in models of immune-related skin diseases, they have been verified in many models of autoimmune disease, a field in which ASCs are likely to be used as a treatment. We expect that these interactions will be examined in models of immune-related skin diseases in the future. In addition, most of the current research on the use of ASCs as a treatment for various disorders is at the experimental stage; large-scale clinical trials should be the ultimate focus of our future efforts.

## Conclusions

ASCs directly or indirectly alleviate immune-related skin diseases via direct interaction with immune cells and paracrine mechanisms. ASCs affect immune cells involved in both the innate and adaptive immune responses to inhibit their pro-inflammatory functions. ASCs also mediate immune pathways by secreting cytokines, growth factors, anti-inflammatory mediators, and active enzymes. Presently, most clinical research focuses on their efficacy and safety in human body, while animal studies are devoted to explored underlying mechanisms of the interaction of ASCs with immune cells and immune factors. The immunomodulatory effect of ASCs has been reported in multiple clinical trials, but these applications need to be further explored and refined, including more standardized preparation methods, higher numbers of patients, and more stringent inclusion criteria. ASC therapy is thus a promising candidate for treatment of immune-related skin diseases and is expected to suppress disease progression and improve patient quality of life.

### Supplementary Information

**Additional file 1: Table S1**. Preclinical studies of the immunomodulatory effect of ASCs in immune-related skin disease. This table summarizes the preclinical applications of the immunomodulatory effect of ASCs in immune-related skin diseases, which helps to understand the recent research directions.

## Data Availability

Not applicable.

## Abbreviations

ASC: Adipose-derived stem cell
MSC: Mesenchymal stem cell
DC: Dendritic cell
NK cell: Natural killer cell
EV: Extracellular vesicle
BM-MSCs: Bone marrow mesenchymal stem cells
Treg cell: Regulatory T cell
Th1: Type 1 helper T cell
Th2: Type 2 helper T cell
Th17: Type 17 helper T cell
CTL: Cytotoxic T lymphocyte
Breg cell: Regulatory B cell
IL: Interleukin
TGF: Transforming growth factor
MLRs: Mixed lymphocyte reactions
HGF: Hepatocyte growth factor
VEGF: Vascular endothelial growth factor
PGE_2_: Prostaglandin E_2_
TSG-6: Tumor necrosis factor alpha induced protein-6
IDO: Indoleamine-pyrrole 2,3,-dioxygenase
NOS: Nitric oxide synthase
Arg: Arginase
CM: Conditioned medium
Exos: Exosomes
AD: Atopic dermatitis
PBMC: Peripheral blood mononuclear cell
SLE: Systemic lupus erythematosus
TNF-*α*: Tumor necrosis factor-*α*
SSc: Systemic scleroderma
IFN-*γ*: Interferon-*γ*
Exos: Exosomes
Scl-GvHD: Chronic sclerodermatous graft-versus-host disease
SVF: Stromal vascular fraction
hSVF: Human stromal vascular fraction
hASCs: Human adipose-derived stem cells
mASCs: Mouse adipose-derived stem cells
dASCs: Dog adipose-derived stem cells
rASCs: Rat adipose-derived stem cells
*α*-SMA: *α*-Smooth muscle actin
MMP1: Matrix metalloproteinase 1
TIMP1: Tissue inhibitor of metalloproteinase-1 protein
COX-2: Cyclo-oxygenase
dcSSC: Diffuse cutaneous SSc
PRP: Platelet-rich plasma
