# Supplementary material for: Adipose-derived stem cells in immune-related skin disease: a review of current research and underlying mechanisms
Source: Stem Cell Res Ther. 2024 Feb 8;15:37. doi: 10.1186/s13287-023-03561-8 (PMC10854049; doi:10.1186/s13287-023-03561-8)
Supplement: Supplementary file 1 — Additional file 1: Table S1. Preclinical studies of the immunomodulatory effect of ASCs in immune-related skin disease. This table summarizes the preclinical applications of the immunomodulatory effect of ASCs in immune-related skin diseases, which helps to understand the recent research directions. [file 13287_2023_3561_MOESM1_ESM.docx]

**Table S1 Preclinical studies of the immunomodulatory effect of ASCs in immune-related skin disease**

| Diseases | Model | Source | Route | Findings and mechanisms | *Ref* |
| --- | --- | --- | --- | --- | --- |
| SSc | Nude mice | hSVF | Subcutaneous injection | Correction of dermal sclerosis and increased local vascularization. | ^96^ |
|  | BALB/c mice | hASCs | Intravenous injection | Decreased skin fibrosis via reducing inflammatory cytokines and enhancing the MMP1/TIMP1. | ^97^ |
|  | Nude mice | hASCs | Lipotransfer | Improved skin texture by suppression of TGF-β1 and collagen expression. | ^98^ |
|  | C57BL/6 mice | mASCs | Intravenous  injection | Attenuation of skin fibrosis via suppressed infiltration of T cells and macrophages, and inhibited IL-6 and IL-13. | ^89^ |
|  | In vitro | hASCs/hASC-EVs | Co-culture | Antifibrotic and pro-remodeling effects via decreased collagen and upregulated MMP1/TIMP1. | ^99^ |
|  | Nude mice | ASC-enriched lipotransfer | Lesion injection | Reversal sclerosis via decreased apoptotic cells and inflammation in skin. | ^100^ |
|  | Nude mice | mASCs | Subcutaneous injection | Attenuated skin fibrosis and improved fat retention via suppressed skin inflammation and promoted angiogenesis, and enhanced adipogenesis via the AKT/ERK signaling pathway. | ^101^ |
| SLE | In vitro | hASCs | Co-culture | Suppression of the number and capability of T_H_17 lymphocytes in active lupus. | ^102^ |
|  | (NZB×NZW)F1 mice | hASCs | Intravenous injection | Alleviation of SLE via increased IL-10 levels and numbers of T_reg_ cells, and restoration of cytokine production. | ^103^ |
|  | Roquin(san/san) mice | hASCs | Intravenous injection | Suppression of autoimmunity via induction of B_reg_ cell and T_reg_ cell expansion, and decrease effector B cells, T_H_1 cells, and T_H_17 cells. | ^93^ |
|  | MRL/lpr mice | hASCs | Intravenous injection | Inhibition of lupus dermatitis by suppression of the T_H_1/T_H_2 (IFN-γ/IL-4) ratio. | ^80^ |
|  | MRL/lpr mice | mASCs | Intravenous injection | Alleviation of tissue damage by suppressed IL-17 levels and inflammatory cell infiltration. | ^104^ |
|  | MRL-Fas^lpr^/J mice | hASCs | Intravenous injection | Alteration of miRNAs and decreased plasma cell proportion and T_H_1/T_H_2 ratio. | ^105^ |
|  | MRL/lpr mice | mASCs | Intravenous injection | Inhibition of autoimmune progression via decreased T_H_17 cells and increased T_reg_ cells. | ^85^ |
|  | MRL/lpr mice | mASCs | Intravenous injection | Prevention of SLE and previously established disease via suppressed Th17/IL-17-induced inflammation. | ^106^ |
|  | In vitro | hASCs | Co-culture | Suppressed expansion of CD4^+^ and CD8^+^ T cells of SLE patients via upregulation of kynurenines and PGE_2_. | ^22^ |
| AD | Dog | dASCs extract | Applied to the lesion | Alleviation of AD-induced inflammation via contained IL-10 and TGF-β1 | ^107^ |
|  | NC/Nga mice | hASCs | Intravenous injection | Alleviation of AD by regulation of B cell function via COX-2 signaling and suppressed MCs degranulation. | ^108^ |
|  | NC/Nga mice | hASC-Exos | Intravenous or subcutaneous injection | Alleviation of AD by decreased infiltration of MCs and macrophages, and inflammatory cytokines levels. | ^109^ |
|  | BALB/c mice | hASCs/hASC-CM | Intravenous injection | Alleviation of AD by regulation of the MIP-2, miRNA levels, and T_H_1/T_H_2 responses. | ^78^ |
|  | NC/Nga mice | mASCs/mASC-CM | Intralesional injection | Improvement of AD-like skin lesions by decreasing inflammation associated with the T_H_2 immune response and IFN-γ. | ^79^ |
|  | NC/Nga mice | hASC-Exos | Subcutaneous injection | Attenuation of AD-like symptoms via facilitating de novo ceramide synthesis and decreasing inflammatory cytokines expression. | ^110^ |
|  | BALB/c mice | mASCs | Subcutaneous injection | Amelioration of AD by decreased IL-17 secretion of T_H_17 cells. | ^83^ |
|  | BALB/c mice | dASCs/dASC-Exos | Subcutaneous injection | Improvement of inflammation and skin barrier function, and suppression of JAK/STAT signaling. | ^111^ |
|  | Rats | rASCs | Systemic administration | Improvement of AD by decolonization of skin *Staphylococcus aureus* via enhanced phagocytic activity of PBMCs. | ^90^ |
| Psoriasis | C57BL/6 mice | mASCs | Intradermal injection | Inhibition of Psoriatic inflammation via decreased production of T_H_17 cytokines. | ^81^ |
|  | In vitro | hASCs | Co-culture | Inhibition of lymphocytes in Psoriasis by promoting of T_reg_ cell proliferation and decreasing T_H_17 cells. | ^112^ |
|  | C57BL/6 mice | hASCs | Subcutaneous injection | Alleviation of Psoriasis by inhibiting ROS generation, CD45^+^ cell infiltration, and decreasing inflammatory cytokines. | ^82^ |
| Allogeneic skin graft | Rats | rASCs | Injected under the skin graft | Increased skin graft survival by differentiating into endothelial cells and increasing cytokines which enhanced angiogenesis and wound healing. | ^113^ |
|  | CB-17 SCID/mice | hASCs | Injected under the skin graft | Suppression of immune responses in vitro and inhibition of T cell-mediated alloreactivity in vivo via increased function of IDO. | ^114^ |
|  | SD rats | rASCs | Administered at recipient bed | Improved skin graft survival via promoting anti-inflammatory macrophages polarization regulated by decreased NOS levels and increased Arg-1 levels. | ^115^ |
|  | BALB/c mice | mASCs | Intraperitoneal injection | Increased skin allograft survival via decreased IL-6, TNF-α, and lymphocyte abundance. | ^116^ |
| Scl-GvHD | BALB/c mice | hASCs | Intravenous injection | Attenuation skin sclerosis via suppressing collagen expression and infiltration of CD4+ T cells and macrophages. | ^117^ |

*hSVF: Human stromal vascular fraction; hASCs: Human adipose-derived stem cells; mASCs: Mouse adipose-derived stem cells; dASCs: Dog adipose-derived stem cells; rASCs: Rat adipose-derived stem cells; α-SMA: α-smooth muscle actin; MMP1: Matrix metalloproteinase 1; TIMP1: Tissue inhibitor of metalloproteinase-1 protein; COX-2: Cyclo-oxygenase*
